# Didymin Ameliorates Dextran Sulfate Sodium (DSS)-Induced Ulcerative Colitis by Regulating Gut Microbiota and Amino Acid Metabolism in Mice

**DOI:** 10.3390/metabo14100547

**Published:** 2024-10-14

**Authors:** Zhongxing Chu, Zuomin Hu, Feiyan Yang, Yaping Zhou, Yiping Tang, Feijun Luo

**Affiliations:** Hunan Provincial Key Laboratory of Deeply Processing and Quality Control of Cereals and Oils, Hunan Provincial Key Laboratory of Forestry Edible Resources Safety and Processing, National Research Center of Rice Deep Process and Byproducts, Central South University of Forestry and Technology, Changsha 410004, China; 20221100445@csuft.edu.cn (Z.C.); 20220100081@csuft.edu.cn (Z.H.); 20210100085@csuft.edu.cn (F.Y.); t20242821@csuft.edu.cn (Y.Z.); t640194@csuft.edu.cn (Y.T.)

**Keywords:** didymin, ulcerative colitis, inflammation, gut microbiota, amino acid metabolism

## Abstract

**Background:** Didymin is a dietary flavonoid derived from citrus fruits and has been shown to have extensive biological functions, especially anti-inflammatory effects, but its mechanism is unclear. The purpose of this study was to investigate the potential mechanism of didymin that alleviates ulcerative colitis. **Methods and Results**: Our results indicated that didymin could alleviate the symptoms of ulcerative colitis, as it inhibited the expressions of interleukin-6 (IL-6), interleukin-1β (IL-1β) and tumor necrosis factor-α (TNF-α). Didymin also promoted the expressions of claudin-1 and zona occludens-1(ZO-1), which are closely related with restoring colon barrier function. Didymin also increased the abundance of *Firmicutes* and *Verrucomicobiota*, while decreasing the abundance of *Bacteroidota* and *Proteobacteria*. Meanwhile, didymin significantly altered the levels of metabolites related to arginine synthesis and metabolism, and lysine degradation in the colitis mice. Utilizing network pharmacology and molecular docking, our results showed that the metabolites L-ornithine and saccharin could interact with signal transducer and activator of transcription 3 (STAT3) and nuclear factor kappa-B (NF-κB). In this in vitro study, L-ornithine could reduce the expressions of transcription factors STAT3 and NF-κB, and it also inhibited the expressions of IL-6 and IL-1β in the lipopolysaccharides (LPS) induced in RAW264.7 cells, while saccharin had the opposite effect. **Conclusions**: Taken together, didymin can regulate gut microbiota and alter metabolite products, which can modulate STAT3 and NF-κB pathways and inhibit the expressions of inflammatory factors and inflammatory response in the DSS-induced colitis mice.

## 1. Introduction

Inflammatory bowel disease (IBD) is a common chronic and recurrent gastrointestinal disease which can be divided into Crohn’s disease (CD) and colitis according to pathological differences [1]. Colitis is a typical inflammatory bowel disease with a high incidence and brings many problems to people’s lives [2]. Up to now, the cause of colitis has not been fully understood. Many complex key factors affect colitis, including genetics, diet, environment and so on [3]. Clinically, 1,2 steroids, glucocorticoids, aminosalicylic acid and other drugs are commonly used to prevent and treat colitis. However, long-term use of certain drugs may have side effects on the body, especially irreversible damage to the liver and kidney of patients [4]. Therefore, the search for effective and safe drugs for colitis has become a hot spot of clinical research. More and more studies show that dietary polysaccharides, cellulose and flavonoids compounds may have broad prospects in the prevention and treatment of colitis [5].

Didymin (DID) is a flavonoid glycoside found in citrus fruits. According to previous reports, DID has the functions of anti-oxidation, anti-inflammation, protecting the liver and protecting nerves [6]. DID can regulate inflammation-related signaling pathways, such as the MAPK/NF-κB pathway, TLR4/NF-κB pathway and PI3K/AKT pathway, to exert an anti-inflammatory effect, but how DID regulates these signaling pathways is still unclear [7]. Network pharmacology is mainly used to study the interaction between active components and key targets of disease. It is based on the network model of active components–targets–pathways to reveal the mechanisms and potential functions of active components. Molecular docking is an important tool in network pharmacology to predict the binding affinity between compounds and targets [8]. These cutting-edge bioinformatics techniques, in combination with metabolomics, will help to explain the precise molecular mechanisms of DID that alleviate colitis.

In this study, our aim was to evaluate the anti-inflammatory effect of DID and investigate the mechanism of DID alleviating inflammatory in the DSS-induced colitis model. After DID treatment, gut microbiota alteration was analyzed, and we also detected the change in metabolites. Network pharmacology and molecular docking were used to analyze potential metabolites affecting inflammation-related key genes and pathways. An LPS-induced cell inflammatory model was used to prove the potential metabolites, which can modulate inflammation-related pathways and regulate inflammatory factor expressions. Our study aimed to reveal the molecular mechanism of DID that alleviates colitis and provide new clues for the clinical treatment of colitis.

## 2. Materials and Methods

### 2.1. Chemicals and Reagents

DID (CAS Number 14259-47-3, purity ≥ 97%) was purchased from Lemeitian Pharmaceutical Technology Co., Ltd. (Chengdu, China). L-ornithine (CAS Number 70-26-8, purity ≥ 98%) and saccharin (CAS Number 81-07-2, purity ≥ 98%,) were purchased from Naturewill Biotechnology Co., Ltd. (Chengdu, China). MTS was purchased from Promega Co., Ltd. (Madison, WI, USA, Lot No. G358A). DSS (molecular weight, 36-50 kDa; Lot No. M2066; purity ≥ 98%) was purchased from Sigma-Aldrich (St. Louis, MO, USA). LPS from Escherichia coli O127:B8 was purchased from Sigma-Aldrich Co. (St. Louis, MO, USA). Polyclonal antibody TNF-α (Cat. #11948), IL-1β (Cat. #12507), IL-6 (Cat. #12912) and β-Actin (Cat. #12620) were used. The primary antibodies of ZO-1 (Cat. #21773-1-AP) and Claudin-1 (Cat. #13050-1-AP) were purchased from Cell Signaling Technology (Beverly, MA, USA). Anti-mouse IgG HRP conjugate (Cat# W4021) and anti-rabbit IgG HRP conjugate (Cat# V7951) were purchased from Promega Co. (Madison, Wl, USA). Fetal bovine serum (FBS) was purchased from Umedium (Hefei, China), and Dulbecco’s modified essential medium (DMEM) was purchased from Gibco-BRL (Carlsbad, CA, USA). All reagents used in this research were analytical-grade.

### 2.2. Animal and Experimental Design

In order to determine the dose concentration of DID used throughout the experiment, a pre-experiment was carried out before the formal experiment. A total of 15 ICR mice were randomly divided into CON, DSS, 15 mg/kg DID + DSS, 30 mg/kg DID + DSS and 60 mg/kg DID + DSS. The ICR mice (weight, 30.0–35.0 g; male; 8–9 weeks old) were provided by the Hunan Prima Pharmaceutical Research Center Co., Ltd. (Liuyang, Changsha, Hunan, China). The mice were fed under 24 ± 2 °C, 65 ± 5% humidity, 12/12 h dark/light cycles and <50 dB noise. The mice were fed a normal diet and freely drank during the experiment.

We determined the lavage concentration of DID and then conducted the formal experiment. There was a 6-day adaptive period before the normal experiment. And then, 30 mice were randomly divided into 3 groups (CON, DSS, and DSS + DID). Each group contained 10 mice. The CON-group mice were provided with pure water for 2 weeks. The DSS-group mice were provided with drinking water supplemented with 2% (*w*/*v*) DSS for 14 days. The mice in the DID group were provided with a DID suspension with water (30 mg/kg/day) for 14 days. They were also provided with drinking water supplemented with 2% (*w*/*v*) DSS for 14 days. The ethical regulations of Hunan Province regarding animal experiments and guidelines for the care and use of laboratory animals were followed in this study. The use of animals and experimental agreements were approved by the Guidelines for the Care and Use of Experimental Animals, the Hunan Prima Pharmaceutical Research Center Co., Ltd. (HNSE2021(5)069, Liuyang, Changsha, Hunan, China).

### 2.3. Disease Activity Index (DAI) Evaluation

For every day of the entire experiment, the mental state and vitality, water intake, food intake, weight change, stool consistency and total blood loss of the mice were monitored. And the DAI score was composed of weight loss, blood in stool and stool characteristics, the details of which are referred to in our previous paper. For the determination methods of stool characteristics and occult blood, refer to the paper by [9]. Before the ending of the final experiment, all mice were fasted overnight, their weight was measured and they were killed by deep anesthesia with isoflurane. After the colon and rectum proximal separation, we measured the ileum cecum junction by using the length between the proximal colon and rectum. The weight was also measured. Rectal feces and cecal contents were collected. A portion of the tissue needed to be fixed, and the rest of the tissue was frozen with liquid nitrogen and stored in a refrigerator at −80 °C.

### 2.4. Colon Histopathology Observation

Mid-colon tissues were harvested and fixed in 10% formalin solution for 24 h, followed by dehydration in graded ethanol solution. Tissue sections were immersed in molten paraffin. We then embedded the colon tissue into a slicing machine and cut it into 4–5-micron sections; adsorption took place on the clean glass, and then the sections were dried overnight at 36–37 °C. Colon tissue dewaxing was performed with xylene reagents, and then we used graded ethanol solvent dehydration. We subjected the colon tissue-section line to hematoxylin–eosin (H&E) staining, and using future-proof microscope (push around Inc., Tokyo, Japan) observation, we checked the fossae gland for the presence of colon epithelial cells, neutrophils and lymphocytes infiltrating density.

### 2.5. Protein Extraction and Western Blot Analysis

The middle part of the colon was taken and crushed in liquid nitrogen. The crushed protein samples were collected and homogenized on ice. RIPA lysis buffer, 1% PMSF, 1% cocktail and 1% phosphatase inhibitors were added. After mixing, it was incubated at 4 °C for 1 h and centrifuged at 13 × 1000 rpm for 16 min to remove the supernatant. We added 2 × SDS loading buffer and boiled at 100 °C for 15 min to fully denature the protein. The denatured protein mixture underwent electrophoresis on an 8.0–15.0% SDS-PAGE gel, which was then transferred to a PVDF membrane. TBST solution containing 5.0% bovine serum albumin (BSA) was closed at 25 °C for 2 h. We then incubated overnight with a suitable concentration of primary antibody at 4 °C. After that, we washed with TBST film washing solution 3 times at 25 °C for 10 min each time. Then, the appropriate concentration of secondary antibodies was incubated at 25 °C for 1 h. Also, we again washed the samples with TBST film washing solution 3 times, 10 min each time. Finally, immunoreactive proteins were detected using the ECL Plus Western blot assay (Pierce, Rockford, IL, USA) and imprinted in a gel imaging system (ChemiDoc-XRS+, BIO-RAD) [10].

### 2.6. RNA Extraction and Quantitative Real-Time PCR Analysis

We then took the required sample tissue and ground it in a mortar pre-cooled with liquid nitrogen. Total RNA was extracted by TransZol-Up (TransGen Biotech Co., Ltd., Beijing, China). After analyzing the quality and concentration of the RNA and testing the integrity of the RNA, single-strand cDNA was synthetized by using 1.2 μg total RNA and HiScript IV RT SuperMix for qPCR (+gDNA wiper) (R423-01, Vazyme Biotech Co., Ltd., Nanjing, Jiangsu, China). A real-time PCR system (CFX96, Applied Biosystems Co., Ltd., Waltham, MA, USA) was used for RT-qPCR. The amplification conditions were 94 °C, initial denaturation for 3 min; 94 °C, denaturation for 30 s; 60 °C, annealing for 40 s; and 72 °C, extension for 1 min, 40 cycles. Relative expression levels of the target genes were calculated based on the 2^−ΔΔCt^ (RQ) method. Please refer to the previous article for details of the experiment [11].

### 2.7. DNA Sequencing and Gut Microbiota Analysis

Total DNA of bacteria was extracted and purified from colon contents (*n* = 6 per group) according to the manufacturer’s instructions. PCR product was identified with 2.0% agarose gel, extracted and further purified with the Axy Prep DNA gel extraction kit (Axygen, Union City, CA, USA). Purified amplicon was quantitatively sequenced using QuantiFluor™-ST (Promega, Madison, WI, USA) on the Illumina MiSeq platform according to a standard protocol (Majorbio Corporation, Shanghai, China). The results were analyzed using the Majorbio cloud platform (https://www.MajorBio.com).

### 2.8. Non-Target Metabolomics Assay of Serum Metabolites

According to the requirements of sample preparation, 100.0 μL serum samples (n = 6 per group) were collected for metabolomics analysis. Metabolome profile was measured using a UHPLC-QTOF-MS system (AB Sciex Pte. Ltd., Milford, MA, USA). Using Progensis QI software (Waters Co., Milford, MA, USA), we determined the baseline data of the original filter, peak recognition, and integral and performed a retention-time correction and peak-alignment analysis. On this basis, data matrices for retention time, mass-to-charge ratio and peak intensity were obtained. Progensis QI was used to identify metabolites and suitable criteria based on mass cleavage (MS/MS analysis). We used HMDB (http://www.hmdb.ca) and Metlin (https://metlin.scripps.edu). We analyzed the chemical structure and name of metabolites and then used the MajorBio Cloud Platform (https://www.majorbio.com). PCoA, a free online platform, uses principal coordinate analysis to assess overall differences between groups. SIMCA and MetaboAnalyst 5.0 were used for data analysis and pathway enrichment analysis. In addition, Spearman correlation analysis was used to assess the correlation between the differences in gut microbiota and the composition of variable metabolites in each group.

### 2.9. Selection of Representative Serum Metabolites

According to the VIP value and *p*-value (VIP > 3; *p* < 0.01), representative serum metabolites were screened. L-ornithine is related to arginine synthesis and metabolism. Lysine degradation product is saccharin. L-ornithine and saccharin were used to study the network pharmacological analysis.

### 2.10. Network Pharmacological Analysis

#### 2.10.1. Collection and Screening of Active Components of Drugs and Prediction of Targets

We used SwissTargetPrediction (http://www.swisstargetprediction.ch/, accessed on 30 July 2024) to collect saccharin and L-ornithine target protein and the corresponding gene abbreviation.

#### 2.10.2. Collection of Disease-Related Genes

In the Gene Cards database (https://www.genecards.org/), OMIM database (https://www.omim.org/) and Disgent database (https://www.disgenet.org/), we input “Inflammation”; we then searched, collated and merged the results and eliminated duplicate values.

#### 2.10.3. Intersection of Drug and Disease Target

We input the results obtained from 2.10.1 and 2.10.2 into Venny2.1 (https://bioinfogp.cnb.csic.es/, accessed on 30 July 2024). According to the matching process, the results automatically overlap between target genes, pointing to a potential target of drugs to treat disease.

#### 2.10.4. Data Visualization and Construction of PPI Network

The intersection targets obtained in 2.10.3 were imported into the STRING database (https://cn.string-db.org/), and the “Organism” was selected as “Homo sapiens”, with the highest confidence score ≥ 0.4, and the free targets were eliminated. The targets in the network were used as the core targets for drug treatment of the disease, and the results were saved in the form of TSV and imported into Cytoscape 3.9.1 software for analysis. We used the “Generate Style from Statistics” in Tools to set the network, and the node (Degree) size and color gradient corresponded to the Degree value.

#### 2.10.5. GO Enrichment Analysis and KEGG Pathway Enrichment Analysis

The potential core targets of drugs for disease treatment were imported into the DAVID database, and the species “H. sapiens” was selected to set the threshold < 0.01 as the setting condition. GO and KEGG pathway enrichment analyses were performed respectively, and the analysis report was saved and sorted. Through the microscopic drawings (http://www.bioinformatics.com.cn/), the website outputted the results.

### 2.11. Molecular Docking

Molecular-docking technology mainly uses the knowledge of physical chemistry, metrology and other cross-disciplines to study the geometric structure and interaction force between molecules. Molecular docking is a commonly used tool to study the interaction between target proteins and active ingredients. The semi-flexible docking method was used throughout the docking process [12]. Firstly, the protein structure was processed by removing water molecules, adding polar hydrogen atoms and calculating the point charge. Protein-based Receptor files that met the docking requirements were generated using Receptor Grid Generation. We needed to set the appropriate size of the docking area box. Ligand structures were drawn using 2D Sketcher and processed using Lig prep plates. The docking procedure was performed using standard precision molecular docking (SP docking). For detailed procedures and parameters, please refer to previous research [13]. The results obtained from the docking were visualized using Maestro software. The stability and interactions of these complexes were analyzed in depth.

### 2.12. In Vitro Study of Metabolites in Serum

In order to study the effects of serum metabolites on the body, the expressions of inflammatory factors in cells after LPS induction were investigated by in vitro experiments. RAW 264.7 cells (Mouse mononuclear macrophage leukemia cells) were provided by BioHermes Co., Ltd. (Wuxi, China). RAW264.7 cells were cultured in DMEM medium containing 10% FBS and 1% penicillin–streptomycin. The cell culture environment was 37 °C, with 70% humidity and 5% CO_2_. L-ornithine and saccharin were dissolved in the DMEM base and filtered using the former 0.22 μm Millipore membrane. Then, 6–8 × 10^4^ mL/well RAW 264.7 cells were inoculated on 96-well plates (100 μL/well), and the used medium was removed after cell adhesion. Culture groups containing 0–100 μg/mL L-ornithine and saccharin were added, respectively, and each concentration was repeated for 3 wells. After 12 h of culture, microphotographs were taken with an inverted microscope (DMI3000 B, Lecia Microsystems CMS GmbH, Mannheim, Germany) for each well, after which 10 μL of MTS was added to each well. After continued incubation for 1 h, an evaluation was performed with a microplate automatic reader (SpectraMax^®^i3x, molecular Devices, LLC., Urstein, Austria) at 490 nm. RAW 264.7 cells were inoculated in an 8 cm petri dish, and the concentration of cells was 1 × 10^7^ cells/mL. They were normal-cultured for 10–12 h. They were then treated with L-ornithine or saccharin for 2 h and then with 1 μg/mL LPS for 1 h. Total proteins were extracted from the cells according to the abovementioned methods, and the expression of related proteins was analyzed by Western blot. Total RNA was extracted from the cells according to the abovementioned methods, and the expressions of genes were analyzed by quantitative real-time PCR.

### 2.13. Statistical Analysis

Software GraphPad Prism9 was used to analyze all statistical analyses. The data of each group were expressed as mean ± standard deviation. Differences among groups were analyzed by one-way ANOVA, followed by Tukey’s post hoc test. A *p*-value < 0.05 was considered statistically significant.

## 3. Results

### 3.1. Determination of the Dose of DID by Gavage

The weight-change rates of the five groups (CON, DSS, 15 mg/kg DID + DSS, 30 mg/kg DID + DSS, and 60 mg/kg DID + DSS) were 37.14%, −14.29%, −8.57%, 2.86% and 2.80%, respectively. The lengths of the colons were 10.13 ± 0.52, 7.21 ± 1.33, 7.49 ± 1.48, 9.11 ± 0.41 and 9.13 ± 0.65 cm, respectively. According to the data of the weight-change rate and colon length of mice, it can be seen that DID had some relief effect on DSS-induced ulcerative colitis. However, the therapeutic effect of the lowest dosage was not obvious. In addition, the dosage of 60 mg/kg DID was twice that of 30 mg/kg, but from the results of the preliminary experiment, its therapeutic effect was not significantly better than that of 30 mg/kg. Therefore, the DID dosage of 30 mg/kg was used in the subsequent formal experiment.

### 3.2. Effects of DID Supplementation on the Colitis Symptoms

**Figure 1:**
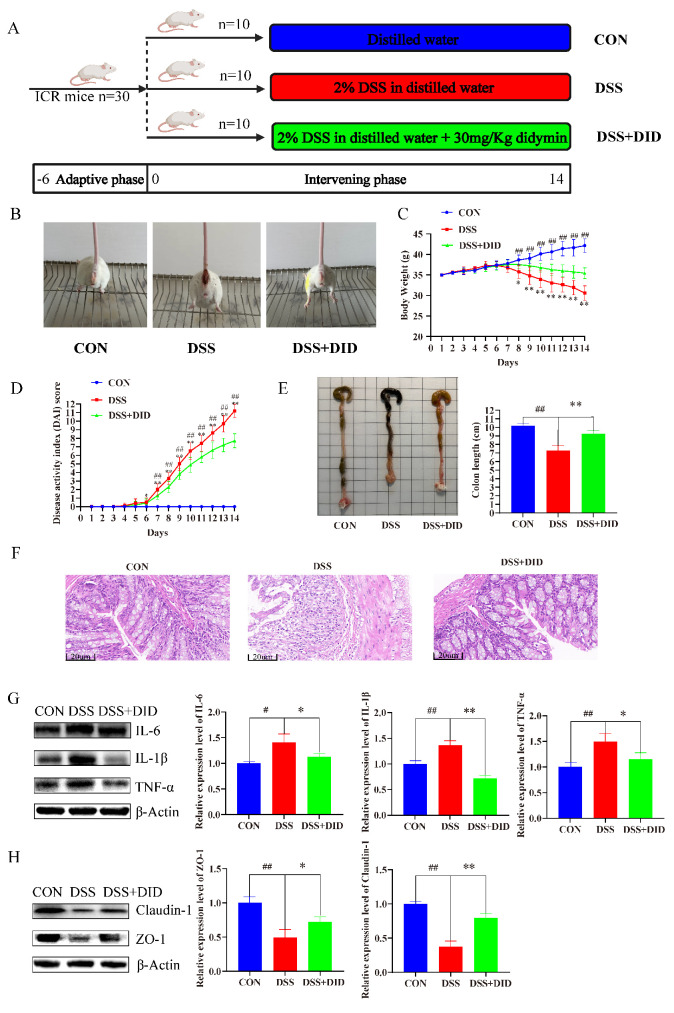
Effect of DID supplementation on the symptoms of DSS-induced UC in mice. (**A**) Animal treatments schedule. (**B**) Bleeding in the colon at the end of the experiment. (**C**) Percentage of the body-weight change during the experiment. (**D**) DAI score. (**E**) Representative image of the colon in different groups and the colon length. Data are presented as means ± SEM, n = 10. (**F**) Representative H&E-stained sections of colonic tissue (20× magnification). (**G**) Inflammation and (**H**) intestinal barrier were analyzed by Western blot. Data are presented as means ± SEM, n = 3. Data are presented as means ± SEM, n = 10. # *p* < 0.05 and ## *p* < 0.01 versus the CON group; * *p* < 0.05 and ** *p* < 0.01 versus the DID group.

In general, the severity of DSS-induced ulcerative colitis was assessed by considering changes in body weight, high or low DAI score, colon length and H&E staining in mice. The schedule of the whole experiment period is shown in Figure 1A. DID alleviated DSS-induced colonic bleeding in mice (Figure 1B). Over the course of the 14-day experiment, the normal-group mice gradually gained weight. However, the DSS-induced mice began to lose weight on days 6 and 7, compared with the control group, but DID could slow the rate of DSS-induced weight loss. In addition, starting from day 8, the difference was statistically significant (*p* < 0.05) (Figure 1C). DAI is an important indicator for evaluating the severity of colitis. The normal-group mice did not develop colitis symptoms. Mice in the DSS group began to develop loose stools or occult blood from day 5, and the symptoms of colitis worsened with the continuous intake of DSS. The DAI score in the DSS group was significantly higher than that in the CON group (*p* < 0.05). DID decreased the DAI score, and the difference was statistically significant compared with DSS group (*p* < 0.01) (Figure 1D). The colon length of the mice was shortened under DSS stimulation, and the DID intervention reversed this phenomenon. DID improved colon length by approximately 30% in colitis mice (Figure 1E). In addition, the histological damage of colon tissue was assessed by H&E staining. The DSS group showed typical features of colitis, with destruction of colon epithelial cells, irregular and incomplete crypt structure and obvious infiltration of inflammatory cells. DID was able to alleviate these reactions and reduce the histological score of the colon (Figure 1F).

To investigate the effects of DID on the expression of inflammatory factors and intestinal barrier protein in colonic tissues of mice with DSS-induced ulcerative colitis, we detected the expressions of IL-6, IL-1β and TNF-α in DSS-stimulated colon tissues via Western blot. DID significantly reduced the protein expression levels of proinflammatory cytokines in the colonic tissues of DSS-induced mice. DID could inhibit the expressions of inflammatory factors IL-6, IL-1β and TNF-α in the colon tissues. The inhibition rates were approximately 10%, 20% and 25%, respectively (Figure 1G). The intestinal barrier function is related to the intercellular tight-binding protein in colon tissue. The intestinal barrier mainly involves the expressions of ZO-1, Claudin-1 and other proteins. By detecting the expression levels of related proteins, it was found that the protein expressions of ZO-1 and Claudin-1 in colon tissues stimulated by DSS were significantly decreased. However, the expression of ZO-1 and Claudin-1 proteins could be restored after DID treatment. DID could recover approximately 50% and 90% of expressions, respectively (Figure 1H). These results indicate that DID plays an important role in inhibiting the expression of inflammatory factors in the colonic tissues of colitis and restoring the intestinal barrier. By detecting the relative expression levels of mRNA for the common inflammatory factors and intestinal barrier protein in colon tissue, we found that DID significantly inhibited the mRNA expression levels of IL-6, IL-1β and TNF-α in the colon tissue of colitis mice, with inhibition rates of 16.34% 17.20% and 17.39%, respectively. DID increased the mRNA expression levels of ZO-1 and Claudin-1, with changes of about 1.05 and 0.69 (Supplementary Figure S1). These results indicated that DID can significantly improve the symptoms of DSS-induced ulcerative colitis.

### 3.3. Effects of DID Supplementation on the Gut Microbiota in Colitis Mice

**Figure 2:**
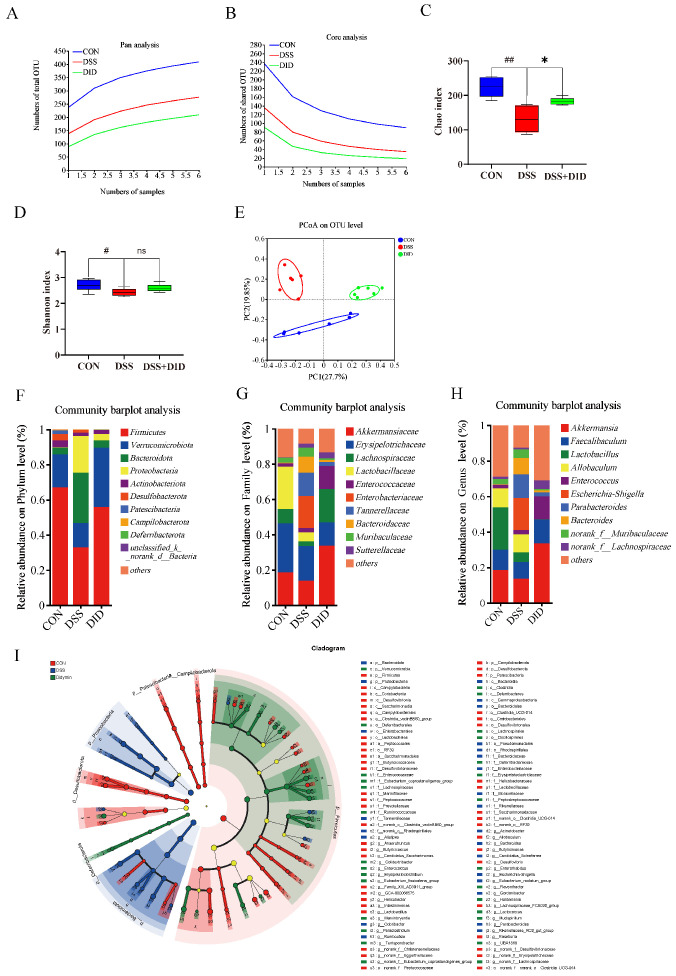
Effects of DID supplementation on the change in the gut microbiota. (**A**) Pan analysis curve. (**B**) Core analysis curve. (**C**) Chao index. (**D**) Shannon index. (**E**) PCoA analysis on OTU level. (**F**) Relative abundances of the gut microbiota at the phylum level. (**G**) Relative abundances of the gut microbiota at the family level. (**H**) Relative abundances of the gut microbiota at the genus level. (**I**) LEfSe multi-level species hierarchical tree diagram, using different colors to represent certain enriched taxa. Data are presented as means ± SEM, n = 6. # *p* < 0.05 and ## *p* < 0.01 versus the CON group; * *p* < 0.05 versus the DID group and ns: there was no significant difference.

To investigate the effects of DID on gut microbiota, bacterial 16S rRNA in fecal contents was analyzed. In the Pan/Core species analysis, an almost-smooth curve could be observed, indicating that the sample size for 16S rRNA sequencing was adequate for the entire experiment (Figure 2A,B). The effects of the DID treatment on the intestinal bacterial diversity in mice were further studied by α- and β-diversity analyses. For the α-diversity analysis, the observed species, Chao index and Shannon index are shown in Figure 2C,D. Compared with the CON group, DSS reduced the observed species-level Chao index (*p* < 0.05) and Shannon index (*p* < 0.01), while the DID treatment revised both indices upwards. PCoA was used to assess β-diversity in the gut microbiota (Figure 2E). Bacteria in the CON group were well separated from bacteria in the DSS group and DID group, and 10 prominent bacteria were detected at the phylum level. Among them, *Firmicutes*, *Verrucomicobiota*, *Bacteroidota* and *Proteobacteria* account for more than 95% of the total gut microbiota (Figure 2F). Under the stimulation of DSS, the abundance of Bacteroidota and Proteobacteria increased significantly, while the abundance of Firmicutes and Verrucomicobiota decreased significantly. DID treatment increased the abundance of Firmicutes and Verrucomicobiota, while it decreased the abundance of Bacteroidota and Proteobacteria. The changes were about 1.6, 3.5, 6 and 8 times, respectively. On the family level, DSS decreased the abundance of Akkermansiaceae and Lachnospiraceae and improved the abundance of Enterobacteroidaceae and Bacteroidaceae. The changes were about 1.8, 3, 15 and 8 times, respectively (Figure 2G). At the genus level, DID was able to change the abundance of Akkermansia, Faecalibaculum, Lactobacillus, Parabacteroides and other genera (Figure 2H). To explore the specific system types of the CON, DSS and DID groups, an LDA > 4 LEfSe analysis was performed. Characteristic bacteria in the CON group include p_Firmicutes, p_Patescibacteria, p_Campilobacterota and p_Desulfobacterot; p_Proteobacteria and p_Bacteroidota were the main Bacteroidota in the DSS group; and p_Verrucomicrobiota was abundant in the DID group (Figure 2I). All of the abovementioned results suggest that supplementation with DID improves the dysregulation of the gut microbiota induced by DSS and, thus, may be responsible for the remission of colitis.

### 3.4. Effect of DID Supplementation on the Serum Metabolites in Colitis Mice

**Figure 3:**
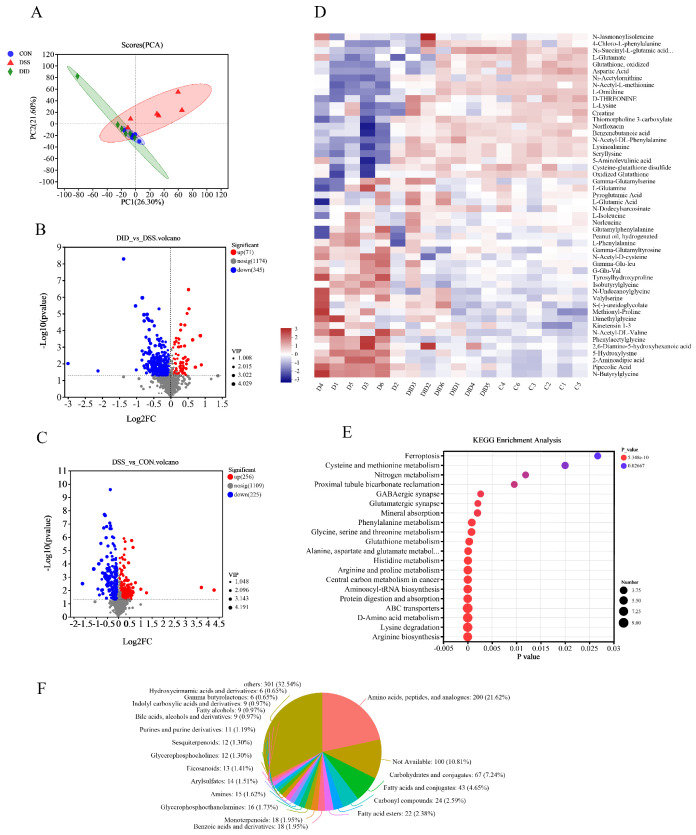
(**A**) Principal component analysis (PCA) of metabolites. (**B**) Volcano plots of DID vs. DSS. (**C**) Volcano plots of DSS vs. CON. (**D**) Heatmap of the metabolites in the CON group altered by DSS responding to DID treatment. (**E**) KEGG pathway enrichment analysis. (**F**) Human Metabolome Database (HMDB) classification. Data are presented as means ± SEM, n = 6.

To assess the metabolic changes induced by DID’s remodeling of the gut microbiota in mice, we used UHPLC-QTOF-MS for non-targeted metabolome profiling of the mouse serum samples. According to the principal component analysis (PCA) model, there was significant metabolite aggregation among the CON group, DSS group and DID group (Figure 3A). The contents of 71 metabolites were increased, and the contents of 345 metabolites were decreased, in the DID group compared with the DSS group. Compared with the CON group, the abundance of 256 metabolites increased and that of 225 metabolites decreased in DSS group (Figure 3B,C). We can see the level changes in different metabolites through the heatmap enrichment analysis, including L-glutamate, glutathione, oxidized, L-lysine, N-acetyl-DL-phenylalanine, D-threonine, lysinoalanine and seryllysine (Figure 3D). The further enrichment analysis of the KEGG pathway in the abovementioned metabolic set showed that DID treatment could induce arginine biosynthesis; glutathione metabolism; alanine, aspartate and glutamate metabolism; arginine and proline metabolism; and aminoacyl-tRNA biosynthesis. And DID treatment could also induce changes in glycine, serine and threonine metabolism; phenylalanine metabolism; and other pathways (*p* < 0.01) (Figure 3E). According to the statistics of 1014 metabolites in the HMDB library, amino acids, peptides and analogues accounted for 21.62%. The proportion of other metabolites was uniform and abundant (Figure 3F). These results suggest that DID supplementation can change amino acid-related metabolism in mice, thus possibly affecting colitis.

### 3.5. Association between Changes in Gut Microbiota and Changes in Amino Acid Metabolism

**Figure 4:**
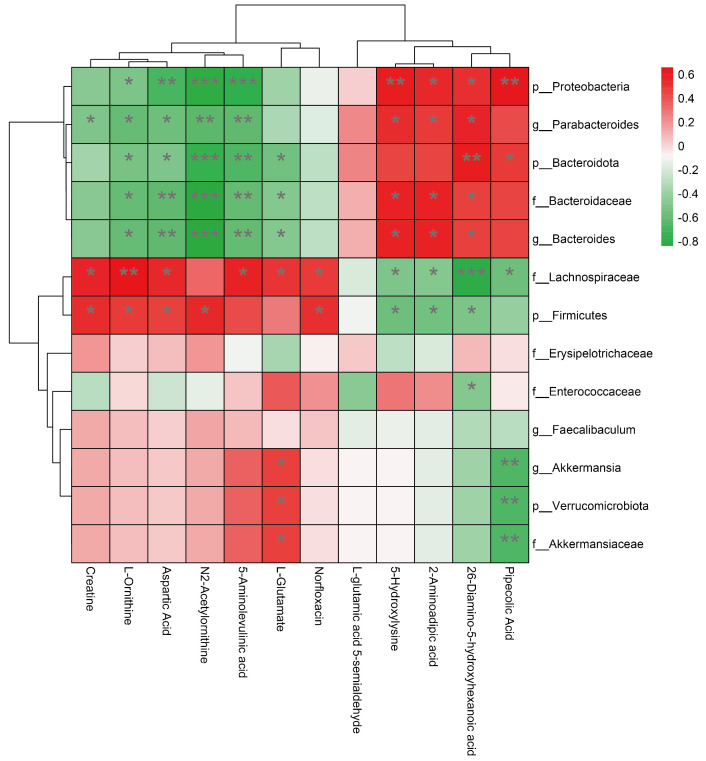
Spearman correlation between gut microbiota and metabolites. * *p* < 0.05, ** *p* < 0.01 and *** *p* < 0.001.

In order to explore the relationship between changes in gut microbiota and changes in amino acid metabolism, we separately studied the changes in gut microbiota and amino acid metabolism caused by DID intervention in a mouse model of colitis. At the phylum level, DID significantly improved the level of Firmicutes and Verrucomicrobiota and reduced the Bacteroidota level. At the family level, DID alleviated the decrease in the abundance of Akkermansiaceae and Lachnospiraceae caused by DSS stimulation and increased the abundance of Enterococcaceae and other gut microbiotas. Similarly, at the genus level, the abundance of Akkermansia, Faecalibaculum, Bacteroides and Enterococcus also changed. According to the results of the blood metabolism test, DID promoted the generation and metabolism of amino acids such as arginine and lysine. In particular, it significantly altered the content level of the related metabolites L-ornithine, N2-acetylornithine, aspartic acid, L-glutamate, N2-succinyl-L-glutamic acid 5-semialdehyde, creatine, 2, 6-diamino-5-hydroxyhexanoic acid, 5-hydroxylysine, L-saccharopine, pipecolic acid, norfloxacin and 5-aminolevulinic acid metabolites (Supplementary Figures S2 and S3). Based on the Spearman analysis, the heatmap showed the correlation between gut microbiota and metabolites. At the phylum level, p__Firmicutes and p__Verrucomicrobiota were positively correlated with metabolites in arginine biosynthesis and arginine metabolism; and p__Proteobacteria and p__Bacteroidota were inversely associated with lysine degradation. At the family level, f__Akkermansiaceae was positively correlated with arginine biosynthesis and arginine metabolism, and it was negatively correlated with lysine degradation. Similarly, f__Erysipelotrichaceae, f__Lachnospiraceae and f__Enterococcaceae were negatively correlated with lysine degradation. However, the positive correlation between the three bacterial groups and arginine biosynthesis and arginine metabolism was not significant. The effects of f__Bacteroidaceae and p__Bacteroidota had similar effects on metabolites. At the genus level, the metabolites affected by g__Akkermansia, g__Faecalibaculum, g__Parabacteroides and g__Bacteroides were almost identical to the metabolites affected at their phylum levels (Figure 4). In conclusion, the protective effect of DID on DSS-induced ulcerative colitis may be related to its regulation of gut microbiota and the host metabolites.

### 3.6. Results of Network Pharmacology Data Analysis

**Figure 5:**
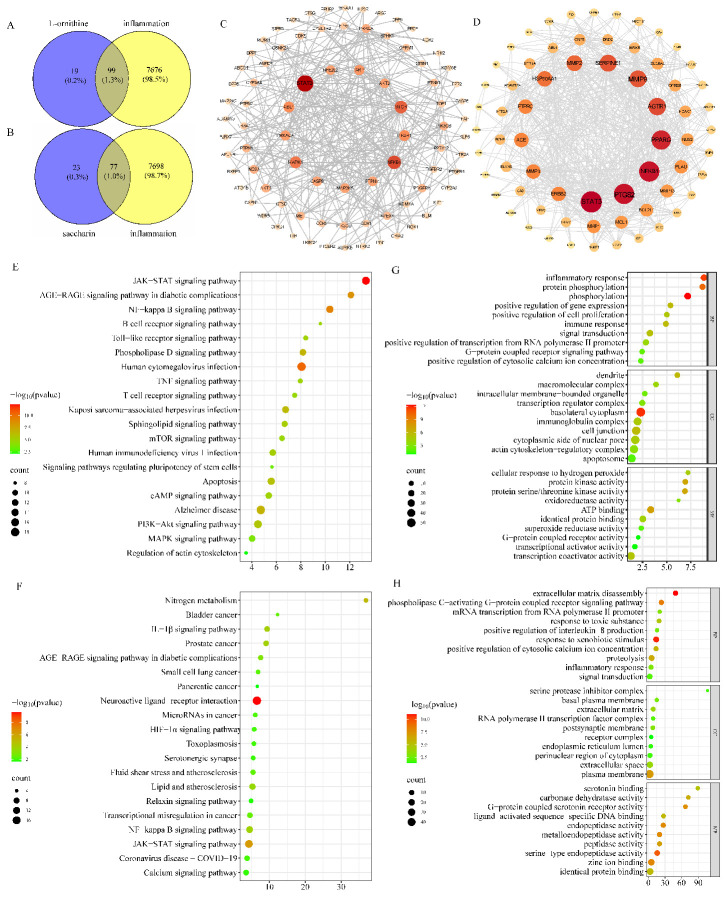
Network pharmacological analysis diagram. (**A**,**B**) Venn diagram analysis of targets of L-ornithine and saccharin with inflammation. (**C**,**D**) Targets of L-ornithine and saccharin targeting inflammation sorted by degree. (**E**) GO analysis of targets of L-ornithine targeting inflammation. (**F**) GO analysis of targets of saccharin targeting inflammation. (**G**) KEGG pathway analysis of targets of L-ornithine targeting inflammation. (**H**) KEGG pathway analysis of targets of saccharin targeting inflammation.

By integrating multiple databases, we identified potential targets for the effects of L-ornithine and saccharin on inflammation. The potential targets of L-ornithine and saccharin are 118 and 100, respectively. There are 7775 potential targets associated with inflammation. The data were processed, and the Venn diagram was generated. The number of core targets related to the inflammation of L-ornithine and saccharin were 99 and 77, respectively (Figure 5A,B). We set the score value > 0.4 to enrich and visualize the data results. The strongest targets of L-ornithine and saccharin were obtained, respectively. The top L-ornithine targets were STAT3, NFKB1, MTOR, MAPK1, PIK3R1, ABL1, PARP1, PRKACA, CASP8, PTPN11, AKT2, NFE2L2 and MAP3K5. The top saccharin targets were STAT3, PTGS2, NFKB1, PPARG, MMP9, AGTR1, SERPINE1, MMP2, HSP90AA1, ERBB2, MMP3, PTPRC and ACE (Figure 5C,D). The application of the GO analysis can effectively promote the identification of biochemical-related functions, thus enhancing the discovery of more identifiable gene-function information. Figure 5E,F show the top 10 words related to the significant enrichment of BP, CC and MF in the GO analysis. The enrichment results showed that L-ornithine was involved in inflammatory response, immune response, signal transduction, transcription regulator complex, immunoglobulin complex, inflammatory response, immune response, signal transduction, transcription regulator complex, immunoglobulin complex, transcriptional activator activity and other biological processes. Saccharin was involved in phospholipase C-activating G-protein-coupled receptor signaling pathway, positive regulation of interleukin-8 production, inflammatory response, response to xenobiotic stimulus, inflammatory response, response to xenobiotic stimulus, RNA polymerase II transcription factor activity, ligand-activated sequence-specific DNA binding and other biological processes. The KEGG pathway analysis was used to retrieve and extract signaling pathways highly associated with L-ornithine, saccharin and inflammation. According to the size of the intersection points, the top 20 most significant (*p* < 0.05) KEGG pathways were selected for visualization (Figure 5G,H). These pathways included the JAK-STAT signaling pathway, IL-1β signaling pathway, NF-κB signaling pathway, B-cell receptor signaling pathway, TNF signaling pathway and other signaling pathways related to inflammation. In summary, based on our network pharmacological analysis, L-ornithine and saccharin may participate in the inflammatory response of the body mainly by affecting STAT3, NF-κB pathway and its downstream genes.

### 3.7. Results of Molecular-Docking Analysis

**Figure 6:**
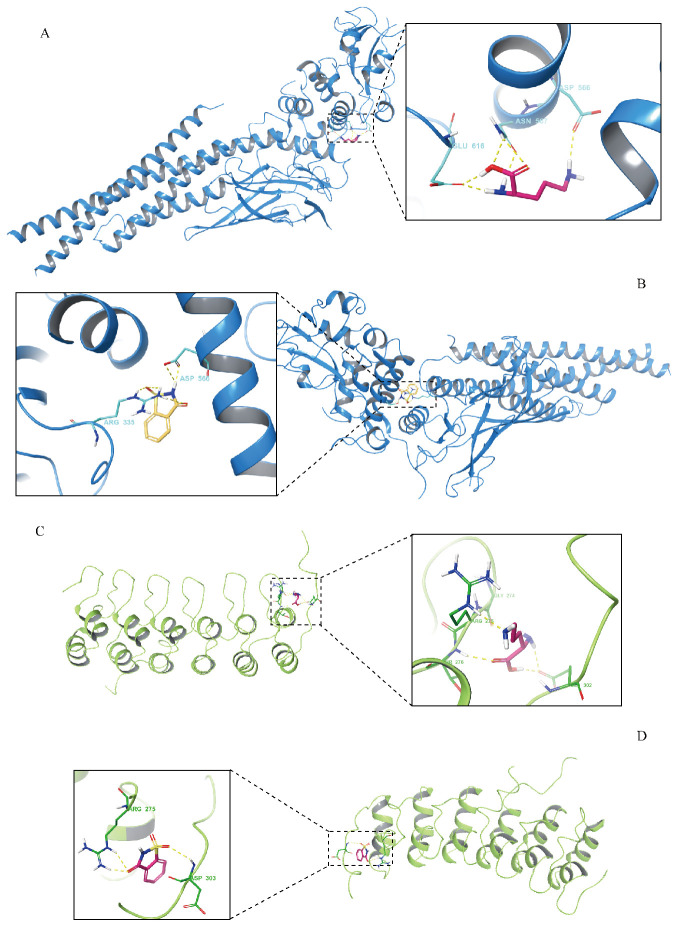
Results of molecular docking. (**A**,**B**) The complex formed by L-ornithine, saccharin and STAT3. (**C**,**D**) The complex formed by L-ornithine, saccharin and NF-κB.

SP docking was performed on L-ornithine and saccharin with STAT3 and NF-κB, respectively. The binding energies of L-ornithine to the two receptor proteins were −3.333 kcal/mol and −4.247 kcal/mol, respectively. The combined energies of saccharin and the two acceptors were −5.573 kcal/mol and −4.913 kcal/mol. Negative binding energy indicated that the compound component could bind to two different receptor proteins. A low negative binding energy indicates a strong binding affinity between the active compound and the target protein. According to the molecular-docking results, L-ornithine and saccharin can bind to STAT3 and NF-κB (Figure 6). Our results suggest that L-ornithine and saccharin may be metabolites that can potentially modulate inflammation-related pathways and may reduce expressions of inflammatory factors.

### 3.8. Evaluated Safety and Dosages of Serum Metabolites

**Figure 7:**
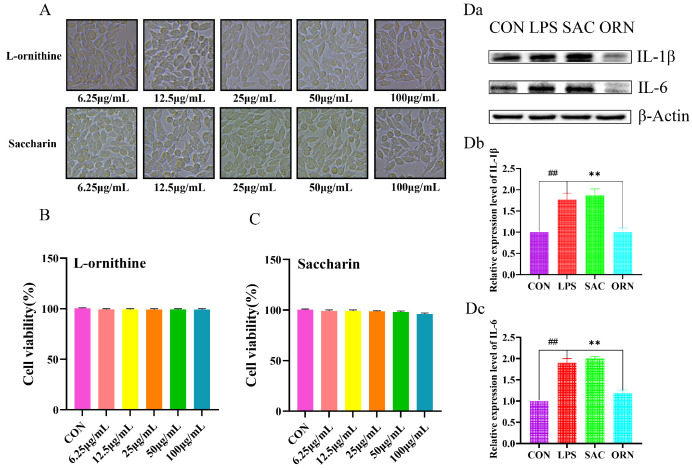
Effects of saccharin and L-ornithine on RAW 264.7 cell viability and LPS-induced inflammatory factor expression. (**A**) Morphological information of RAW 264.7 cells. (**B**,**C**) Cell viability (%). (**D**) Inflammatory factor expressions were analyzed by Western blot. Data are presented as means ± SEM, n = 3. ## *p* < 0.01 versus the CON group; ** *p* < 0.01 versus the ORN group.

L-ornithine, the product, was related to arginine synthesis and metabolism. The lysine degradation products saccharin and L-ornithine were used to investigate the effect on the expressions of inflammatory factors in cells after LPS induction. According to the photos (Figure 7A) and numerical results (Figure 7B,C) of the MTS cell viability assay, the optimal concentration of L-ornithine was determined to be 50 μg/mL, and the concentration of saccharin was 100 μg/mL, which caused cell damage.

### 3.9. Serum Metabolites Affected the Expressions of Inflammatory Factors

The Western blot results showed that saccharin promoted the expressions of IL-6 and IL-1β after LPS induction, while L-ornithine reduced the expressions of IL-6 and IL-1β. The rates of change were approximately 10%, 10%, 50% and 40%, respectively (Figure 7D). The data suggest that L-ornithine has an anti-inflammatory effect and that saccharin has a pro-inflammatory effect, thus matching our results of animal experiments and bioinformatics analysis.

### 3.10. Metabolites Affected mRNA Expressions of Target Genes and Inflammatory Factors

In order to verify the authenticity of the target genes screened by computer-simulation methods such as network pharmacology, molecular docking and molecular dynamics, the mRNA expressions of STAT3 and NF-κB in LPS-induced RAW264.7 cells treated with L-ornithine and saccharin were studied in vitro (Supplementary Figure S4). Also, under 1.0 μg/mL LPS, L-ornithine inhibited the mRNA expressions of STAT3 and NF-κB in RAW264.7 cells. The inhibition rates were 13.09% and 20.73%, respectively. However, saccharin could promote the mRNA expressions of two transcription factors. Compared with the 1.0 μg/mL LPS model group, the change rates of STAT3 and NF-κB transcription factors were 4.44% and 18.18%, respectively. The mRNA expressions of inflammatory factors in LPS-induced RAW264.7 were further detected. Similar to transcription factors, L-ornithine inhibited the mRNA expressions of inflammatory cytokines IL-6, IL-1β and TNF-α at a rate of 17.35%, 35.16% and 16.59%, respectively, compared with the model group. Saccharin reversed this effect, and the change rates of the three inflammatory factors were 15.82%, 16.89% and 7.11%, respectively.

## 4. Discussion

At present, only a few studies have confirmed the role of DID in the treatment of neuroinflammation, ischemic injury, non-alcoholic fatty liver disease and other diseases [14,15,16,17]. Our results also suggest that oral DID could relieve symptoms, such as weight loss and rectal bleeding, caused by DSS-induced ulcerative colitis. DID could effectively reduce the expressions of inflammatory factors in colon tissue, reduce colon inflammatory response, reduce pathological injury of colon tissue and repair the integrity of the intestinal barrier function. Moreover, DID plays a key role in regulating the gut environment and rebalancing the gut microbiota. Compared with the DSS group, DID treatment significantly increases the content levels of microorganisms positively related to the production and metabolism of arginine, such as p_Firmicutes and p_Verrucomicrobiota. However, at present, the specific anti-inflammatory molecular mechanism of DID is not completely clear.

Studies have shown that inhibiting the expressions of inflammatory factors in the host and reducing the level of inflammatory factors in the host are the key ways to alleviate inflammation. TNF-α, IL-1β and IL-6 are important pro-inflammatory cytokines [18,19]. TNF-α can exacerbate inflammatory responses by degrading and destroying host mucous membranes [20]. IL-6 has been shown to increase intestinal tight junction (TJ) permeability by activating the c-Jun N-terminal kinase (JNK) signaling pathway [21]. IL-1β can down-regulate the mRNA level of occludin and increase intestinal TJ permeability [22], all of which are related to the onset and deterioration of colitis symptoms. We investigated the role of DID in alleviating intestinal inflammation and injury severity by detecting the expression levels of IL-6, IL-1β and TNF-α proteins in colon tissue. The results indicate that the addition of DID treatment can reduce the protein levels of inflammatory factors.

The integrity of the intestinal barrier function is essential for intestinal health. Once the intestinal barrier is damaged and destroyed, it is usually accompanied by an increase in intestinal permeability and severe inflammatory response. The TJ protein of intestinal epithelium is involved in maintaining the epithelial barrier function [23]. The destruction of the TJ barrier can cause immune dysregulation, thus inducing intestinal inflammation [24]. Therefore, the expression of TJ protein is widely considered to be the main indicator for assessing the severity of intestinal injury. Compared with the DSS group, DID treatment significantly increased the expression levels of ZO-1 and Claudin-1 in the colon tissue of DSS-induced ulcerative colitis mice. Studies have shown that arginine can protect the intestinal epithelial barrier in rats and IEC-6 cell lines under heat stress [25]. Therefore, the restoration of the intestinal barrier function by DID is likely related to arginine biosynthesis and arginine metabolism.

The gut microbiota plays an important role in regulating the body’s inflammatory response, intestinal integrity and function. The disturbance of the gut microbiota is an important indicator of host pathophysiology [26]. The abundance of gut microbes can be used as an indicator to evaluate the balance and health of the gut [27]. In this study, DID was found to modulate the dysregulation of gut microbiota composition and diversity in DSS-induced ulcerative colitis mice. At the phylum level, DID treatment increased the abundance of Firmicutes and Verrucromicobiota, while it decreased the abundance of Bacteroidota and Proteobacteria. Fábrega et al. reported that the ratio of Firmicutes to Bacteroides in patients with colitis had decreased [28]. Wang et al. proposed that, after DSS treatment, the abundance of Verrucomicobiota had decreased, and the abundance of Proteobacteria had increased, but this phenomenon could be reversed after drug treatment [29]. These results are consistent with our findings. On the family level, DID changed the abundance of Akkermansiaceae, Lachnospiraceae, Enterococcaceae and Bacteroidaceae. On the genus level, Akkermansia, Faecalibaculum, Lactobacillus, Parabacteroides and other genera changed.

Studies have shown that DSS-induced ulcerative colitis is closely related to these microorganisms. Bacteroides, as one of the most abundant anaerobic microorganisms in the human gastrointestinal flora, can decompose a variety of dietary polysaccharides in the host. Most Bacteroides use polysaccharides as a carbon source [30,31]. However, these bacteria also attack and exploit highly glycosylated mucins in the mucous layer, the first defense layer of the intestinal epithelium. When the mucus layer is damaged, the host is more susceptible to immune responses, such as intestinal inflammation caused by harmful metabolites [32]. Moreover, when large numbers of sugar-loving bacteria invade the internal mucus layer, they degrade the glycans and diffuse within the mucus. Thinning the mucous layer causes more bacteria or other microorganisms to penetrate the internal mucous layer and stick to the intestinal epithelium, triggering a cascade of intestinal inflammation that eventually leads to colitis [33,34]. In addition, elevated levels of Proteobacteria can lead to intestinal environmental imbalances and diseases, such as mucosal inflammation, and Proteobacteria can lead to the production of LPS, exacerbating inflammatory bowel disease [35,36]. A fecal flora analysis showed that DSS treatment reduced the diversity of the gut microbiota community. DID can treat gut microbiota dysregulation and dysfunction in mice with DSS-induced ulcerative colitis. The abundance of Proteobacteria was significantly down-regulated. DID increased the relative abundance of Firmicutes and Verrucobacteria and significantly promoted the relative abundance of probiotics such as Akkermansia, Faecalibaculum, etc. These results suggest that it is possible to regulate the microbial composition in the gut and enhance the proliferation of certain bacteria, such as Akkermansia. Its specificity in mucin degradation makes it a key organism at the mucosal interface between gut and host cells. Akkermansia muciniphila is a representative of the verruca microbiome. It tends to colonize widely in the nutrient-rich intestinal mucous layer and plays an important role in maintaining intestinal barrier function, host metabolism and other host biological functions through interactions between gut microbiota and host. Akkermansia muciniphila can also improve DSS-induced ulcerative colitis by regulating some key inflammatory response components, including TNF-α, IL-6 and other pro-inflammatory cytokines [37,38,39].

Serum metabolome is another indicator related to host physiological and pathological processes. Di’Narzo and colleagues found that some serum metabolites were closely related to the severity of inflammation [40]. Our study found that DID can promote the production and metabolism of amino acids, especially L-ornithine, N2-acetylornithine, aspartic acid and L-glutamate, which are related to the production and metabolism of arginine and can inhibit the degradation of lysine. Hydroxylysine, aminoadipic acid and diamino-5-hydroxyhexanoic acid can be increased. Interestingly, the gut microbiota is closely related to blood metabolism. A double-blind randomized controlled clinical trial on obese women found that the regulation of the gut microbiota by probiotics and symbiotic bacteria was related to changes in serum metabolites [41]. Verrucobacteria and Firmicutes showed a positive correlation with arginine, glutamine and 2-oxo-isovalerate, indicating that Verrucobacteria and Firmicutes promote the metabolism of amino acids such as arginine at a certain level, a result that is consistent with our research results. Therefore, DID improves the abundance of Verrucobacteria and Firmicutes and can also accelerate the production and metabolism of amino acids, including arginine, in the body. Similar results occurred in the Wooden breast broiler model [42]. Driuchina and colleagues reported that increased lysine and histidine degradation can be used as biomarkers of high liver fat, and, according to their study, a close relationship between Bacteroides and lysine degradation was found [43]. The saccharopine degradation product saccharin destroys mitochondrial dynamics, leading to mitochondrial damage and, thus, inhibiting the normal growth and development of the body [44]. Mitochondrial saccharin oxidation can lead to fatal mitochondrial damage in body tissues and affect the body’s own repair [45]. At present, the relationship between saccharin and colitis is still full of controversy. After studying the effects of saccharin and other sweeteners on the gut microbiota of the body, it is apparent that sweeteners, including saccharin, can cause gastrointestinal-related metabolic disorders (such as insulin resistance, obesity and inflammation). However, we must consider the influence of factors such as the individual differences of the subjects, such as eating habits, lifestyle, etc. There is no scientific consensus on this conclusion [46,47]. According to our experimental results, DID can reduce the content of saccharin in the serum of colitis mice, and this reduction can reduce the mitochondrial damage of the body to a certain extent and better ensure the energy supply required for the body’s self-repair and growth.

We used network pharmacology to predict the interaction between inflammation-related targets and the potential targets of L-ornithine and saccharin in serum, which are related to amino acid metabolism, and we also used functional and pathway enrichment analyses. The results showed that STAT3 and NF-κ B may be L-ornithine and saccharin targets of the inflammatory reaction. The results of the molecular docking showed that L-ornithine and saccharin could bind closely to the two potential targets. To verify the abovementioned conclusions, we used LPS-induced RAW264.7 as a cellular model of inflammation. MTS was used to screen the appropriate concentration of the two drugs. It is better not to cause a significant influence on cell morphology and cell viability. The experimental data showed that L-ornithine inhibited the expression of two transcription factors, STAT3 and NF-κB. However, saccharin can promote their expression. It verified the forecasting results of network pharmacology and molecular docking. STAT3 and NF-κB, as two important transcription factors, are closely related to inflammatory response. The NF-κB transcription factor and signaling pathway are central coordinators of innate and adaptive immune responses [48]. STAT3 can regulate the expression of a variety of genes related to immune response, thus playing a key role in cellular immune response [49]. STAT3 can be activated by a variety of cytokines, growth factors and oncogenes. Stat3 is also involved in T-cell proliferation induced by IL-2 and IL-6 [50]. The NF-κB and STAT3 are inflammation and immune responses of the two main transcription factors, and their interactions take place on multiple levels. Many cytokines induced by NF-κB or STAT3, such as IL-6, can contribute to STAT3 and NF-κB activation. IL-6 links NF-κB to STAT3 [51]. Cytokines and chemokines can act as mediators of NF-κB activation. TNF-α and IL-6 are two widely studied cytokines whose expression is elevated in a number of different immune responses [29]. STAT3 is also a key factor in the activation of NF-κB, and their interaction contributes to the transcriptional regulation of inflammatory cytokines [52]. Thus, we went on to study the expression of common inflammatory factors in cells. The results showed that L-ornithine and saccharin were able to affect the expression of inflammatory factors in the cells. L-ornithine can alleviate cellular inflammatory response, while saccharin can aggravate this response.

DID treatment can inhibit the abundance of pathogenic bacteria, increase the abundance of potential probiotics and improve the influence of gut microbiota on serum metabolites. Although serum metabolites can affect the inflammatory response through subsequent prediction and verification, due to technical limitations, we cannot accurately know the specific content of relevant metabolites and cannot accurately verify it at the cellular level, which is the disadvantage of the test. Therefore, future research needs cutting-edge technology to accurately quantify relevant metabolites, so as to more accurately study the effects of different content of metabolites on inflammatory response.

## 5. Conclusions

In this study, a DSS-induced colonic inflammation model was used to determine that DID could significantly alleviate the symptoms of colonic inflammation in mice, inhibit the expressions of inflammatory factors in colonic tissues and reduce colonic inflammation. DID could change the intestinal microflora and cause metabolic changes. Using metabolomics and molecular-docking techniques, it was found that DID could promote the synthesis and metabolism of arginine and inhibit the degradation of lysine. DID treatment could regulate the concentrations of L-ornithine and saccharin. In the LPS-induced cell inflammation model, L-ornithine could exert an anti-inflammatory effect, and saccharin promoted an inflammation effect. The results suggest that DID is a potential natural product that can reduce colonic inflammation and damage, and it may be used to prevent and treat colitis, but it lacks clinical investigation in this field; in particular, there are significant differences in the gut microbiota between mice and humans [53]. How DID affects the gut microbiota and metabolites in humans requires further in-depth studies.

## Data Availability

Data will be provided upon request.

## Supplementary Materials

The following supporting information can be downloaded at https://www.mdpi.com/article/10.3390/metabo14100547/s1, Figure S1: Relative mRNA expression level of genes in gut; Figure S2: Representative abundance of gut microbiota; Figure S3: Representative abundance of metabolite; Figure S4: Relative mRNA expression level of genes in RAW 264.7. Table S1: Primers of genes for RT-qPCR analysis.

## Abbreviations

Akt	protein kinase B
CD	Crohn’s disease
DAI	disease activity index
DID	didymin
DMEM	Dulbecco’s modified essential medium
DSS	dextran sulfate sodium
FBS	fetal bovine serum
H&E	hematoxylin–eosin
HPLC	high-performance liquid chromatography
IBD	inflammatory bowel disease
IL-6	interleukin-6
IL-1β	interleukin-1β
JNK	c-Jun N-terminal kinase
LPS	lipopolysaccharide
MAPK	mitogen-activated protein kinases
NF-κB	nuclear factor-kappa B
TJ	tight junction
TLR4	Toll-like receptor 4
TNF-α	tumor necrosis factor-α
PCoA	principal coordinate analysis
PCR	polymerase chain reaction
PI3K	phosphatidyl inositol 3-kinase
PMSF	phenylmethanesulfonyl fluoride
RIPA	radioimmunoprecipitation assay buffer
RT	retention time
STAT3	signal transducer and activator of transcription 3
SDS-PAGE	sodium dodecyl sulfate–polyacrylamide gel electrophoresis
ZO-1	zonula occludens protein 1
