# Supplementary material for: Didymin Ameliorates Dextran Sulfate Sodium (DSS)-Induced Ulcerative Colitis by Regulating Gut Microbiota and Amino Acid Metabolism in Mice
Source: Metabolites. 2024 Oct 14;14(10):547. doi: 10.3390/metabo14100547 (PMC11509612; doi:10.3390/metabo14100547)
Supplement: Supplementary file 1 [file metabolites-14-00547-s001.zip › metabolites-3208204-supplementary.pdf]

Supplementary materials

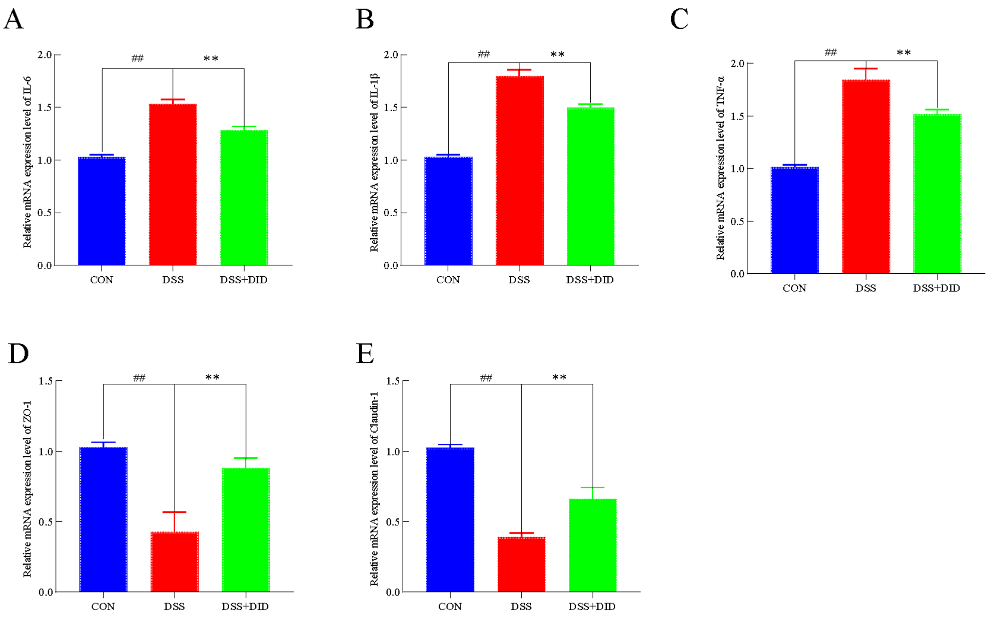

Supplementary Figure S1. (A-C) Relative mRNA expression level of inflammatory factor; (D-E) Relative mRNA expression level of intestinal barrier protein. Data are presented as means ± SEM, n = 3. #: $p < 0.05$ and ##: $p < 0.01$ versus the CON group; *: $p < 0.05$ and **: $p < 0.01$ versus the DID group.

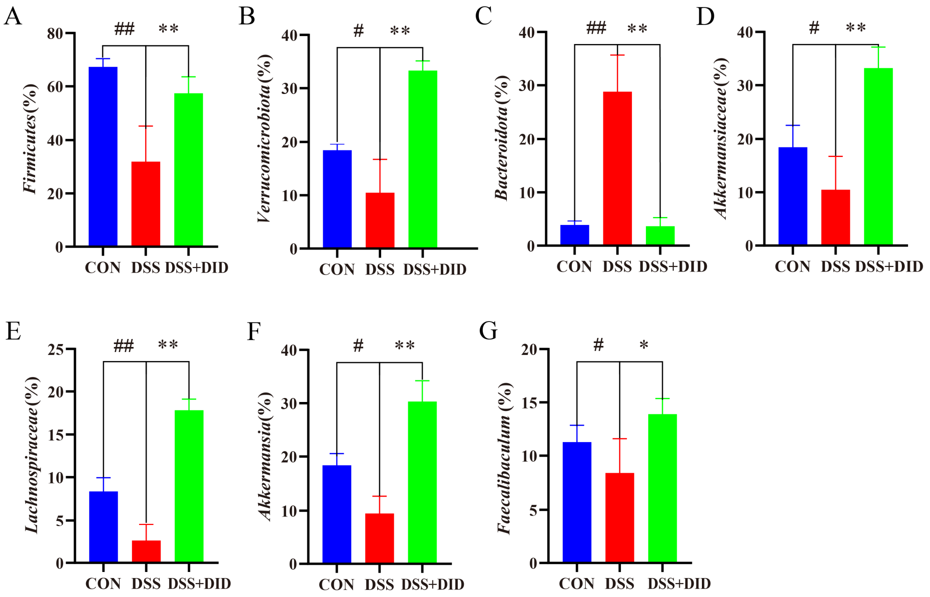

Supplementary Figure S2. Representative abundance of gut microbiota . (A)

Firmicutes abundance; (B) *Verrucomicrobiota* abundance; (C) *Bacteroidota* abundance; (D) *Akkermansiaceae* abundance; (E) *Lachnospiraceae* abundance; (F) *Akkermansia* abundance; (G) *Faecalibaculum* abundance. Data are presented as means ± SEM, n = 6. #: $p < 0.05$ and ##: $p < 0.01$ versus the CON group; *: $p < 0.05$ and **: $p < 0.01$ versus the DID group.

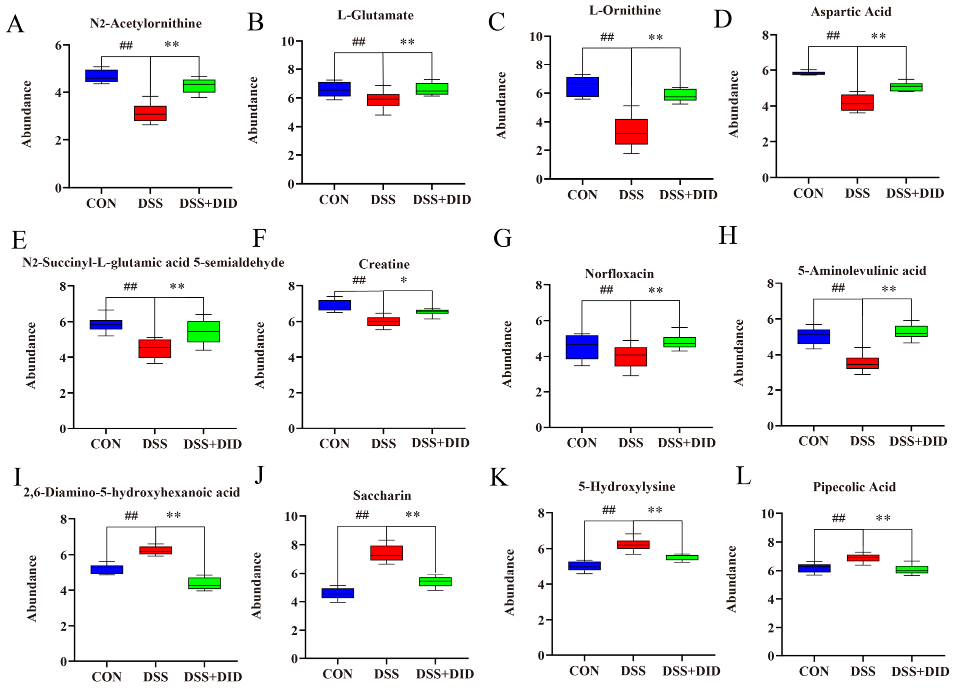

Supplementary Figure S3. Representative abundance of metabolite. (A) N_2-acetylornithine abundance; (B) L-glutamate abundance; (C) L-ornithine aundance; (D) Aspartic acid abundance; (E) N_2-succinyl-L-glutamic acid 5-semialdehyde abundance; (F) Creatine abundance; (G) Norfloxacin abundance; (H) 5-Aminolevulinic acid abundance; (I) 2,6-Diamino-5-hydroxyhexanoic acid abundance; (J) Saccharin abundance; (K) 5-hydroxylysine abundance; (L) Pipecolic acid abundance. Data are

presented as means ± SEM, n = 6. #: $p < 0.05$ and ##: $p < 0.01$ versus the CON group; *: $p < 0.05$ and **: $p < 0.01$ versus the DID group.

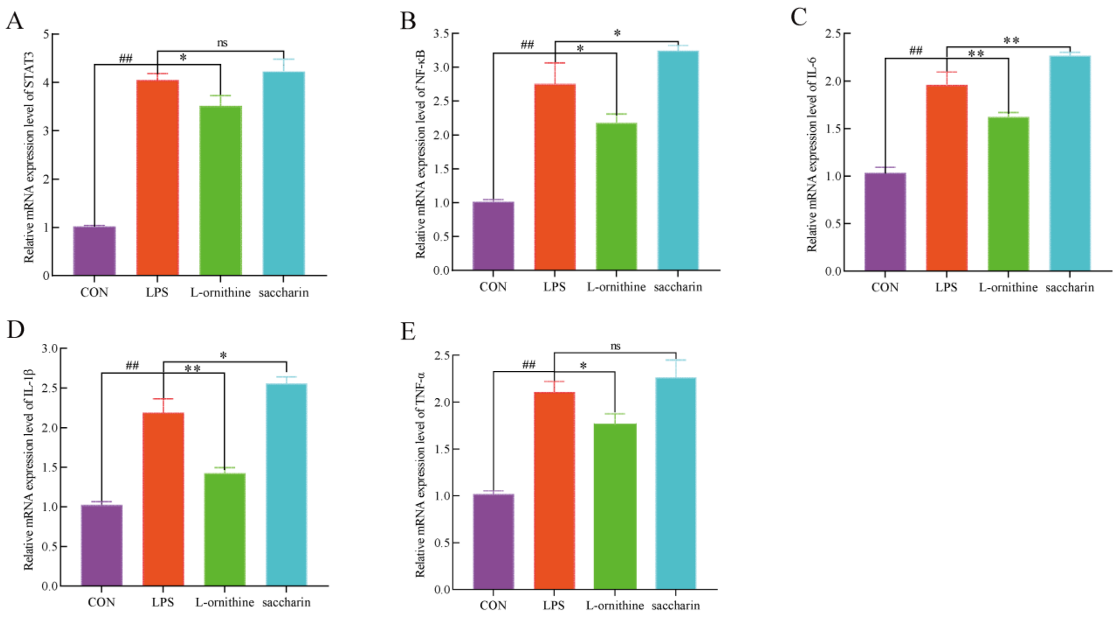

Supplementary Figure S4. (A-B) The relative mRNA expression of STAT3 and NF-κB in RAW264.7; (C-E) The relative mRNA expression inflammatory factor in RAW 264.7. Data are presented as means ± SEM, n = 3. #: $p < 0.05$ and ##: $p < 0.01$ versus the CON group; *: $p < 0.05$ and **: $p < 0.01$ versus the ORN group.

Supplementary Table S1. Primers of genes for RT-qPCR analysis.

Gene name	Sense (5’-3’)	Anti-sense (5’-3’)
STAT3	TGT CTC CAC TTG TCT ACC T	TGT CTC CAC TTG TCT ACC T
NF-κB	CAA GAG TGA TGA CGA GGA G	CAA GAG TGA TGA CGA GGA G
IL-6	ATG GAT GCT ACC AAA CTG GAT	TGA AGG ACT CTG GCT TTG TCT
IL-1β	GAG CAC CTT CTT TTC CTT CAT CTT	TCA CAC ACC AGC AGG TTA TCA TC
TNF-α	CAA AAT TCG AGT GAC AAG CCT G	GAG ATC CAT GCC GTT GGC
ZO-1	CCT TCT GAT GGT GCT CTG	CCT TCT GAT GGT GCT CTG
Claudin-1	CTT CAG CAG AGC AAG GTT	CTT CAG CAG AGC AAG GTT
β-Actin	CCA TAA ACG ATG CCG GA	CAC CAC CCA TAG AAT CAA GA
